# Prevalence of radiologically isolated syndrome in a pediatric population-based cohort: A longitudinal description of a rare diagnosis

**DOI:** 10.1177/1352458521989220

**Published:** 2021-01-22

**Authors:** CL de Mol, AL Bruijstens, PR Jansen, MHG Dremmen, YYM Wong, A van der Lugt, TJH White, RF Neuteboom

**Affiliations:** Department of Neurology, MS Center ErasMS, Erasmus MC University Medical Center Rotterdam, Rotterdam, The Netherlands The Generation R Study Group, Erasmus MC University Medical Center Rotterdam, Rotterdam, The Netherlands; Department of Neurology, MS Center ErasMS, Erasmus MC University Medical Center Rotterdam, Rotterdam, The Netherlands; Department of Complex Trait Genetics, Center for Neurogenomics and Cognitive Research, Amsterdam Neuroscience, Amsterdam UMC, Amsterdam, The Netherlands Department of Clinical Genetics, Amsterdam UMC, Amsterdam, The Netherlands; The Generation R Study Group, Erasmus MC University Medical Center Rotterdam, Rotterdam, The Netherlands Department of Radiology and Nuclear Medicine, Erasmus MC University Medical Center Rotterdam, Rotterdam, The Netherlands; Department of Neurology, MS Center ErasMS, Erasmus MC University Medical Center Rotterdam, Rotterdam, The Netherlands; Department of Radiology and Nuclear Medicine, Erasmus MC University Medical Center Rotterdam, Rotterdam, The Netherlands; Department of Child and Adolescent Psychiatry, Erasmus MC University Medical Center Rotterdam, Rotterdam, The Netherlands Department of Radiology and Nuclear Medicine, Erasmus MC University Medical Center Rotterdam, Rotterdam, The Netherlands; Department of Neurology, MS Center ErasMS, Erasmus MC University Medical Center Rotterdam, Rotterdam, The Netherlands

**Keywords:** Radiologically isolated syndrome, epidemiology, prevalence, pediatrics, case reports, multiple sclerosis

## Abstract

**Background:**

Radiologically isolated syndrome (RIS) is typified by multiple sclerosis (MS)-like lesions on imaging, without clinical MS symptoms. The prevalence of pediatric RIS is largely unknown.

**Objective:**

The objective of the study is to provide an estimated RIS prevalence in a population-based cohort of children.

**Methods:**

We used data from the Generation R study to identify the childhood RIS prevalence.

**Results:**

In 5238 participants, only one RIS case was identified (prevalence: 0.02%; 95% confidence interval (CI): 0.00–0.11). During a 62-month follow-up, imaging examinations showed accrual of new focal demyelinating lesions; however, no clinical MS symptoms occurred.

**Conclusions:**

This study shows that the occurrence of RIS in children from the general population is rare.

## Introduction

Radiologically isolated syndrome (RIS) is defined as the presence of demyelinating lesions, suggestive of multiple sclerosis (MS) without occurrence of clinical MS symptoms.^1^ It is reported in 0.1%–0.7% of adults who underwent brain magnetic resonance imaging (MRI) for complaints not typically compatible with MS (e.g. migraine).^2^ Within 5–10 years, between one-third and half of RIS cases are diagnosed with MS, with children showing earlier fulfillment of the diagnostic criteria.^3–5^ Although knowledge of RIS in children is increasing and specific pediatric diagnostic criteria have been proposed, data on RIS prevalence in childhood remain scarce.^4,6,7^

Here, we provide information on pediatric RIS prevalence using a large population-based birth cohort study and describe the follow-up of identified cases.

## Methods

For the current study, we investigated MRI data from children enrolled in the Generation R Study.^8^ Three waves of MRI examinations were performed within this population-based cohort: phase 1: a subgroup of children between the ages of 6 and 10,^9^ the whole study group in phase 2: children around 9 years,^10^ and phase 3: children around 13 years. Participants were imaged with a 3T MRI scanner: the first subgroup (6–10 years) with an MR750 Discovery MRI scanner and the other two groups (around 9 and 13 years) with an MR750w Discovery scanner (General Electric, Milwaukee, WI, USA). The imaging protocol encompassed, among others, a coronal 3-dimensional (3D) T_1_-weighted sequence, sagittal 3D T_2_-weighted sequence, and axial spin-echo diffusion-weighted sequence. No gadolinium was administered due to the population-based design of the study. Incidental findings were rated by a team of researchers and neuroradiologists as previously described.^11^ RIS was assessed with adult Okuda criteria and pediatric criteria proposed by the PARIS consortium.^1,4^

Parents or legal representatives provided written informed consent of all study participants within the Generation R study. Identified RIS cases provided additional informed consent for the usage of clinical data. The Medical Ethical Committee of the Erasmus Medical Center approved the study protocol.

## Results

After excluding overlapping subjects, 5238 participants had MRI scans of sufficient quality to be rated for incidental findings. Participants’ descriptive characteristics of different waves are shown in Table 1.

**Table 1.** Participants’ descriptive characteristics throughout the Generation R MRI study waves.

	Phase 1 (*n* = 1070)	Phase 2 (*n* = 4092)	Phase 3 (*n* = 3545)	Total^a^ (*N* = 5238)
Age at scan, years, median (IQR)	7.96 (7.08–8.57)	9.94 (9.76–10.29)	13.82 (13.58–14.27)	NA
Male, *n* (%)	572 (53.5)	2036 (49.8)	1699 (47.9)	2592 (49.5)
Reported ethnicity				
Dutch	726 (67.9)	2398 (58.6)	2137 (60.3)	3061 (58.4)
Western	78 (7.3)	358 (8.7)	322 (9.1)	465 (8.9)
Non-Western	266 (24.9)	1250 (30.5)	1019 (28.7)	1598 (30.5)
Unknown	0 (0.0)	86 (2.1)	67 (1.9)	114 (2.2)
Presence of maternal MS, *n* (%)	1/939 (0.11)	6/3344 (0.18)	4/2956 (0.14)	6/4485 (0.13)
Presence of paternal MS, *n* (%)	1/744 (0.13)	3/2674 (0.11)	2/2404 (0.08)	3/3477 (0.09)
EBV-seropositivity, *n* (%)	378/742 (50.9)	1274/2624 (48.6)	1138/2308 (49.3)	1695/3344 (50.7)
Serum vitamin D levels, nmol/L, median (IQR)	65.0 (45.1–82.9)	67.0 (49.0–83.1)	66.0 (48.4–82.4)	65.7 (47.9–82.0)
RIS cases identified, *n*	0	1	0	1
Observed RIS prevalence, % (95% CI)	0.000 (NA)	0.024 (0.00–0.13)	0.000 (NA)	0.019 (0.00–0.11)

IQR: interquartile range; NA: not applicable; MS: multiple sclerosis; RIS: radiologically isolated syndrome; CI: confidence interval; EBV: Epstein–Barr virus.

a Non-overlapping subjects from phases 1, 2, and 3.

One participant showed white matter abnormalities fulfilling the adult Okuda and proposed pediatric PARIS criteria for RIS.^1,4^ This resulted in a general RIS prevalence of 0.019% (95% confidence interval (CI): 0.00–0.11) and a wave-specific prevalence of 0.024% (95% CI: 0.00–0.13) between the ages of 9 and 11 years (phase 2; Table 1).

**Figure 1.**
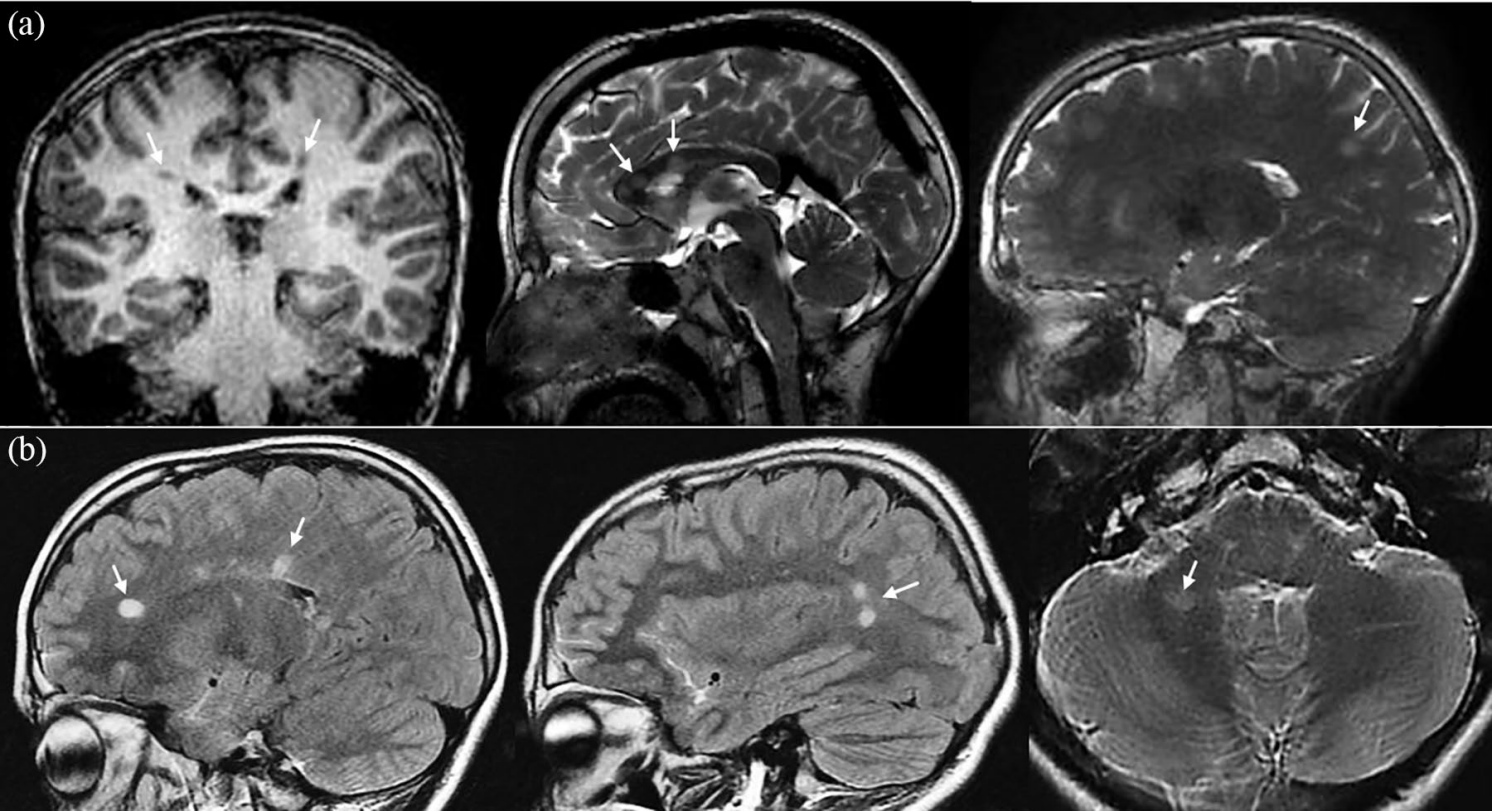
MR images at baseline and follow-up. (a) One coronal T_1_-weighted and two sagittal T_2_-weighted MR images from the brain imaging protocol of the Generation R Study, belonging to the 11-year-old identified male RIS case. The coronal image shows periventricular T_1_-hypointense white matter lesions in both the right and left parietal lobe. The sagittal T_2_-weighted images demonstrate additional intracallosal and subcortical T_2_-hyperintense white matter lesions. (b) Sagittal T_2_-weighted fluid-attenuated inversion recovery (FLAIR) and axial T_2_-weighted MR images of the same patient at follow-up brain imaging (22 months later). The T_2_ FLAIR sagittal images show hyperintense lesions in the periventricular white matter of the supratentorial brain. These white matter lesions were new in comparison with the previous baseline MR examination. The axial T_2_-weighted image shows the infratentorial hyperintense white matter lesion in the right cerebellar hemisphere.

The boy described above was scanned at the age of 11. His first MRI scan showed multiple (>9) well-circumscribed white matter lesions, including several periventricular lesions, intracallosal lesions, and an infratentorial lesion, in addition to T1-hypointense lesions with unknown gadolinium enhancement status (Figure 1).

This Dutch patient (Moroccan descent) was examined at the Dutch pediatric MS center at the age of 12. At the time of the first scan, he had no history of clinical events. However, just 2 months prior to the clinical assessment, he experienced a vertigo episode for a maximum of 7 days. No clinical care was sought out at the time of the symptoms, and at the moment of examination, these had fully recovered. During neurological assessment, no abnormalities were identified; Expanded Disability Status Scale score was 0, urological assessment, including uro-flowmetry, and visual evoked potential examination were normal. A new clinical MRI scan shortly after this clinical assessment, 22 months after the first scan, showed new white matter lesions, but no gadolinium enhancement (Figure 1). No new infratentorial lesions were observed that could account for the vertigo episode. Additional spinal cord MRI showed several cervical lesions. Further investigations showed no indication for other diagnoses, including negative blood test results for aquaporin-4 and myelin oligodendrocyte glycoprotein antibodies. Through genotyping, the patient was found to have heterozygosity of HLA-DRB1*15:01. There was evidence of a remote Epstein–Barr virus infection (serum IgG antibodies against EBNA1 and VCA) and vitamin D level in serum was low (31 nmol/L, normal reference: 50–120 nmol/L).

Follow-up clinical MRI scans showed new lesions 1 and 3 years after the first clinical assessment, including gadolinium enhancement. At the time of last follow-up, 62 months, the patient had not experienced any clinical event. Till now, no immunomodulatory treatment has been started.

## Discussion

In this study, we show that the RIS prevalence in a cohort of developing children between the ages of 6 and 16 is low (0.02%). This is in line with another study in a pediatric MRI cohort of 833 participants that also observed only one patient with a suspected demyelinating lesion, although this patient appeared not to fulfill the Okuda and PARIS criteria for RIS.^12^

Compared with the reported prevalence of adult RIS, our observed prevalence of pediatric RIS is low.^2^ This difference in prevalence could be due to the population-based approach in our study and the younger age of our participants. Another possibility is that our reported prevalence might be an underestimation of the RIS prevalence, as no T_2_ fluid-attenuated inversion recovery sequence was performed within the Generation R Study, which is optimal for the detection of white matter lesions. Another limitation to our study is that while we provide an overall prevalence of RIS between ages 6 and 16, the majority of our participants was 10 years or older. We could therefore have been underpowered to detect possible RIS in this younger age group. Nevertheless, the effect of this relative underrepresentation of children aged between 6 and 10 years on the overall RIS prevalence is expected to be limited as pediatric RIS is typically diagnosed at a higher age.^4,7^

In our study, we did not observe the previously reported female overrepresentation in (pediatric) RIS.^2,4,7^ Next to cohort size, this may be due to the even sex distribution in the Generation R study, based on its population-based inclusion.^8^ This could have made our study relatively underpowered to detect the known female overrepresentation in RIS.

Compared with the general Dutch population, our study had a relative overrepresentation of non-Western children, due to the multi-ethnic Generation R cohort.^8^ This may have influenced our results, as we have previously observed a higher prevalence of pediatric onset of MS in non-Western children in the Netherlands.^13^ The described patient had not experienced any history of relapsing-remitting clinical symptoms at the time of initial MRI scan and was therefore diagnosed with RIS. Whether or not disease modifying therapy should be started in RIS patients with new MRI lesions, without clinical neurological events, is controversial.^6^ The subsequent vertigo episode was not objectified, and the second MRI scan did not show explanatory lesions for this possible clinical episode. Although debatable, we chose a close monitoring policy instead of starting immunomodulatory treatment.

To conclude, we observed that prevalence of RIS in a population-based cohort of children is low. As prevalence appears to be lower compared with adults, extrapolation of information from adult studies on RIS to children may not apply. Therefore, standardized follow-up in those rare children with RIS is needed to increase knowledge on the clinical management of these children. Finally, our study shows that pediatric population–based studies on risk factors for RIS and MS would require considerable numbers of participants.
